# Oxidized cholesterol as the driving force behind the development of Alzheimer’s disease

**DOI:** 10.3389/fnagi.2015.00119

**Published:** 2015-06-19

**Authors:** Paola Gamba, Gabriella Testa, Simona Gargiulo, Erica Staurenghi, Giuseppe Poli, Gabriella Leonarduzzi

**Affiliations:** Department of Clinical and Biological Sciences, School of Medicine, University of TurinOrbassano, Torino, Italy

**Keywords:** Alzheimer’s disease, oxidative stress, inflammation, oxidized cholesterol, oxysterols

## Abstract

Alzheimer’s disease (AD), the most common neurodegenerative disorder associated with dementia, is typified by the pathological accumulation of amyloid Aβ peptides and neurofibrillary tangles (NFT) within the brain. Considerable evidence indicates that many events contribute to AD progression, including oxidative stress, inflammation, and altered cholesterol metabolism. The brain’s high lipid content makes it particularly vulnerable to oxidative species, with the consequent enhancement of lipid peroxidation and cholesterol oxidation, and the subsequent formation of end products, mainly 4-hydroxynonenal and oxysterols, respectively from the two processes. The chronic inflammatory events observed in the AD brain include activation of microglia and astrocytes, together with enhancement of inflammatory molecule and free radical release. Along with glial cells, neurons themselves have been found to contribute to neuroinflammation in the AD brain, by serving as sources of inflammatory mediators. Oxidative stress is intimately associated with neuroinflammation, and a vicious circle has been found to connect oxidative stress and inflammation in AD. Alongside oxidative stress and inflammation, altered cholesterol metabolism and hypercholesterolemia also significantly contribute to neuronal damage and to progression of AD. Increasing evidence is now consolidating the hypothesis that oxidized cholesterol is the driving force behind the development of AD, and that oxysterols are the link connecting the disease to altered cholesterol metabolism in the brain and hypercholesterolemia; this is because of the ability of oxysterols, unlike cholesterol, to cross the blood brain barrier (BBB). The key role of oxysterols in AD pathogenesis has been strongly supported by research pointing to their involvement in modulating neuroinflammation, Aβ accumulation, and cell death. This review highlights the key role played by cholesterol and oxysterols in the brain in AD pathogenesis.

## Introduction

Alzheimer’s disease (AD) is the most common age-related neurodegenerative disorder associated with dementia, and the progressive deterioration of mental capacities. It is a complex disease characterized by progressive memory impairment, cognitive deficit, and personality changes; these symptoms are due to substantial synaptic and neuronal loss occuring in specific brain areas, especially in the neocortex, hippocampus, and other subcortical regions. Brains from AD patients show distinct neuropathological features, which we now know are the hallmarks of the disease: extracellular deposits of amyloid β (Aβ) peptides in the form of senile plaques, Aβ deposits in the cerebral blood vessels, and intracellular inclusion of neurofibrillary tangles (NFT) composed of hyperphosphorylated tau protein (Querfurth and LaFerla, 2010; Chopra et al., 2011).

One of the events that promotes AD pathogenesis is the abnormal processing of amyloid precursor protein (APP), which leads to excess production of Aβ peptides through the sequential enzymatic actions of beta-site APP cleaving enzyme 1 (BACE1), a β-secretase, and γ-secretase, both enzymes of the amiloidogenic pathway. An imbalance between the production and clearance of Aβ peptides in the brain, and their aggregation, cause Aβ to accumulate, with subsequent formation of senile plaques. Depending on the site of γ-secretase cleavage, two major forms of Aβ are generated: a peptide of 40 amino acids (Aβ_1–40_), and one of 42 amino acids (Aβ_1–42_). Aβ_1–42_ is the predominant species of Aβ in senile plaques, and the insoluble oligomers and intermediate amyloids are its most neurotoxic forms (Walsh and Selkoe, 2007). Aβ oligomers are hypothesized to cause neuronal damage and cognitive failure by generating free radicals, as well as mitochondrial oxidative damage, synaptic failure, and inflammatory changes in AD brains (Oddo et al., 2003; Mattson, 2004; Reddy and Beal, 2005; Castellani et al., 2010). Different assembly states of Aβ, and its accumulation in different cellular compartments, can then affect critical pathways, thereby facilitating the development of tau pathology. Experimental evidence confirms that Aβ accumulation precedes and drives NFT formation (Götz et al., 2001; Lewis et al., 2001).

Besides aging, which is the most obvious risk factor for the disease, a number of theories point to other risk factors, such as culture, lifestyle, head injury, and genetics. Other risk factors are associated with vascular disease, including hypercholesterolemia, hypertension, atherosclerosis, coronary heart disease, smoking, obesity, and diabetes (Mayeux, 2003). Some evidence suggests that the dietary intake of homocysteine-related vitamins (vitamin B12 and folate), antioxidants (vitamins C and E), unsaturated fatty acids, and also a moderate alcohol intake, especially in the form of wine, may reduce the risk of AD (Luchsinger and Mayeux, 2004).

Although environmental factors increase the risk of AD, this disease can also be caused by various gene mutations (Gatz et al., 2006). In this contest, mutation in the genes *APP*, presenilin 1 (*PSEN1*), and presenilin 2 (*PSEN2*) accounts for most cases of the familial (or early-onset) form of AD, by increasing the production and aggregation of Aβ and amyloid plaque formation (Tanzi and Bertram, 2005). Conversely, the apolipoprotein E (*ApoE*) gene is an important genetic risk factor for the sporadic (or late-onset) form of AD (Raber et al., 2004). The contribution of other candidate genes is probably less important, and none has been verified. A possible explanation for this difficulty in clarifying the genetic background might be that the sporadic form of AD is not a uniform disease entity, and that several susceptibility-enhancing genes may act in concert, each conferring only a small increase in risk, in a complex interaction with environmental factors.

However, although the pathophysiology of AD is still not clearly understood, considerable evidence indicates that many events participate in the development and progression of the disease, including oxidative stress, inflammation, glial cell activation, dysregulation of metal ions and calcium, presence of ApoEε4, altered cholesterol metabolism, and dysregulation of intercellular communication among brain cells (Quintanilla et al., 2012).

## Oxidative Stress in AD Pathogenesis

It has been extensively reported that free radicals are pathologically important in neurodegenerative diseases, and that the brain tissue is exposed to oxidative damage during the development of AD, already from its early onset (Smith et al., 2000, 2010; Mariani et al., 2005; Zhu et al., 2005; Reynolds et al., 2007). Because age is a significant risk factor for AD, it is also widely accepted that oxidative stress increases with age leading to the accumulation of oxidative damage in biomolecules (Butterfield and Kanski, 2001; Martin and Grotewiel, 2006; Jacob et al., 2013).

The brain is particularly vulnerable to oxidative damage for numerous reasons, but chiefly because it utilizes about 25% of the respired oxygen, with a consequent increase of free radicals; it also contains high concentrations of catalytic iron and lipids, which are easily oxidized by free radicals. Further, the brain contains relatively low levels of antioxidants and antioxidant defense enzymes, and is thus not very efficient at removing free radicals (Ansari and Scheff, 2010; Mazzetti et al., 2015). Because the brain has a high lipid content, it is extremely vulnerable to oxidative species, with the consequent enhancement of lipid peroxidation and cholesterol oxidation, and the subsequent formation of end products, mainly 4-hydroxynonenal and oxysterols, respectively from the two processes (Sottero et al., 2009; Reed, 2011).

Within the brain, neurons are the cells most vulnerable to excess reactive oxygen species (ROS) and reactive nitrose species (RNS), and their survival depends on the antioxidant action of astrocytes. Astrocytes are very important for normal brain function, because of their ability to actively promote neuroprotection, in particular by releasing glutathione, which protects neurons from oxidative stress (Shih et al., 2003). However, neurons can also defend themselves through an intrinsic mechanism of antioxidant defense involving the glucose metabolism (Fernandez-Fernandez et al., 2012).

AD brains display high levels of oxidative stress, and a direct association between free radical generation and the presence of Aβ plaques has been shown both in living AD mouse models and in human AD tissue (McLellan et al., 2003). However, it is still not clear whether oxidative stress is a cause or a consequence of the neuropathology associated with AD (Zhu et al., 2007; Bonda et al., 2010; Smith et al., 2010; Luque-Contreras et al., 2014). In support of its being a cause, it has been proposed that oxidative stress precedes the onset of clinical and pathological AD symptoms, including Aβ deposition, NFT formation, vascular malfunction, and cognitive decline (Nunomura et al., 2001; Praticò et al., 2001). In this connection, in a triple-transgenic mouse model of AD, mimicking AD progression in humans, the levels of antioxidants (glutathione and vitamin E) were found to be decreased, and the extent of lipid peroxidation increased, before the appearance of senile plaques and NFT (Resende et al., 2008). Moreover, as a prominent early event, oxidative stress is believed to contribute to tau hyperphosphorylation in neurons (Su et al., 2010) and, of note, it has been observed that neuritic and cored amyloid plaques show evidence of oxidatively modified Aβ (Head et al., 2002). Conversely, Aβ is a potent promoter of oxidative stress, since it is a potent generator of both ROS (Ding et al., 2007) and RNS (Combs et al., 2001). Within the Aβ sequence, it has been suggested that methionine 35 plays an important role in promoting oxidative activity. When this amino acid is replaced by cysteine, the oxidative stress induced by Aβ is greatly attenuated (Butterfield and Boyd-Kimball, 2005; Butterfield et al., 2013). In this connection, it has also been proposed that the amyloid oligomers may insert themselves into the lipid bilayer, causing lipid peroxidation and, consequently, oxidative damage to proteins and other biomolecules (Butterfield et al., 2001). Aβ oligomers are, indeed, the strongest inducers of oxidative stress among all Aβ species (Tamagno et al., 2006; Naylor et al., 2008). Generation of free radicals, altered membrane properties, as well as disturbed calcium homeostasis, may also underlie the apoptotic effect of Aβ oligomers (Malaplate-Armand et al., 2006). It has also been observed, not only in cultured neurons but also *in vivo* using the double transgenic model of AD (APP/PS1), that Aβ causes an increase in oxidative stress that leads to phosphorylation of p38, which in turn phosphorylates tau at its T231 residue (Giraldo et al., 2014).

Mitochondrial dysfunction is another feature of AD pathogenesis (Castellani et al., 2002). Defects in the mitochondria are typically defects of the electron transport chain; these contribute both to the hyperproduction of a variety of ROS, and to the deficiency of several key enzymes responsible for oxidative metabolism that, in turn, cause cell damage and eventual death (Cottrell et al., 2001). Moreover, it has been shown that oxidative species, through mitochondrial impairment, cause tau hyperphosphorylation leading to neuron and synapse loss (Melov et al., 2007). Mitochondrial dysfunction and oxidative damage have been investigated in triple-transgenic mice that develop both Aβ and tau disorders. These mice exhibited increased oxidative stress, manifested by increased hydrogen peroxide production and lipid peroxidation (Yao et al., 2009; Reddy, 2011).

APP and Aβ have also been associated with dysfunctional consequences for mitochondrial homeostasis and cell death (Manczak et al., 2006). APP impairs mitochondrial energy metabolism, thus causing mitochondrial abnormalities leading to ROS production (Anandatheerthavarada et al., 2003); accumulation of APP in the mitochondrial import channel then potentially inhibits mitochondrial import (Reddy et al., 2004). Aβ is also considered a potent mitochondrial poison, especially affecting the synaptic pool. It appears that Aβ enters the mitochondria, induces free radical generation, disrupts the electron transport chain, and ultimately causes mitochondrial dysfunction (Mungarro-Menchaca et al., 2002). Aβ, then, inhibits key mitochondrial enzymes in the brain and in isolated mitochondria (Caspersen et al., 2005; Reddy and Beal, 2008), and cytochrome c oxidase is also specifically attacked (Crouch et al., 2005). A recent study on the transgenic mouse brain confirmed that Aβ accumulates in neuronal mitochondria, thus affecting mitochondrial function, as shown by increased mitochondrial permeability, the decline of both respiratory function and cytochrome c oxidase activity, and increased mitochondrial oxidative stress (Du et al., 2010).

An association between ApoEε4 and oxidative stress-mediated damage in AD has been also suggested. Despite playing a beneficial role, by maintaining lipid homeostasis and redox balance, ApoE can also contribute to oxidative damage in an isoform-dependent manner, the ApoEε4 isoform being the most harmful in AD (Luque-Contreras et al., 2014). The *ApoEε4* genotype is also involved in mitochondrial dysfunction (Chang et al., 2005), and might be a risk for potential antioxidant system loss in AD (Shea et al., 2002).

**Figure 1:**
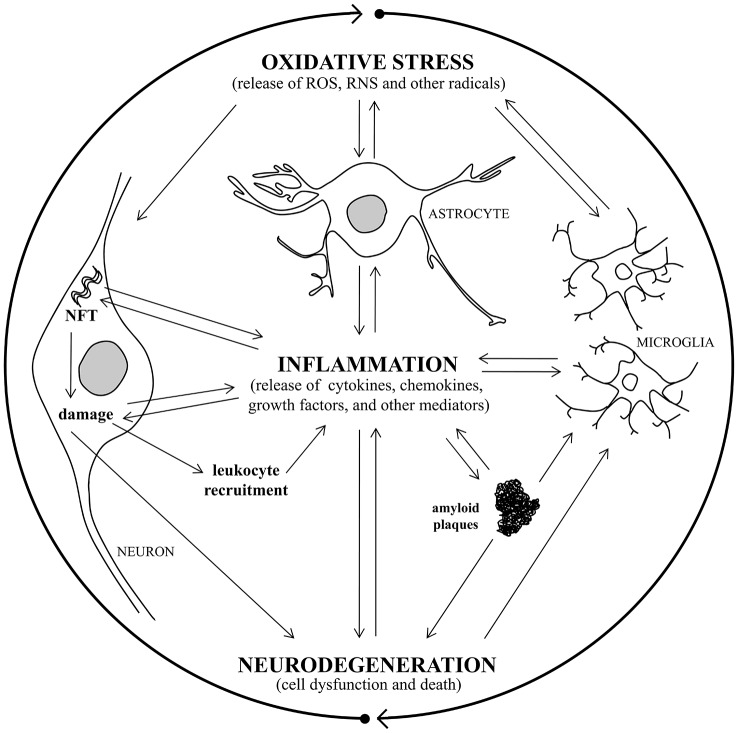
**A vicious circle connects oxidative stress, inflammation, and neurodegeneration in Alzheimer’s disease (AD)**. Oxidative stress damage and inflammatory response are closely associated with AD, causing neurodegeneration. Oxidative stress induces activation of microglia and astrocytes with a consequent increase of pro-inflammatory mediator production and, in turn, glial activation leads to toxic radical release, exacerbating neuronal damage. Consequently, the resultant cellular damage amplifies the inflammatory response, with glial activation and leukocyte recruitment, leading to further inflammation in the AD brain. The release of inflammatory cytokines leads to amyloid plaque and neurofibrillary tangle (NFT) formation, that triggers inflammatory molecule release and causes neuronal damage, with consequent neurodegeneration.

Alterations in cerebrovascular regulation have recently been ascribed to the early stages of AD, and the vascular endothelium is also a target for oxidative stress leading to endothelium dysfunction (Iadecola, 2004; Park et al., 2008). Chronic hypoperfusion may thus play an important role in the pathophysiology of AD, because it induces oxidative stress, and over time this damage could initiate mitochondrial failure (Sochocka et al., 2013). A recent study has shown that inhibition of NADPH oxidase activity can mitigate cognitive impairment in rodent models of hypoperfusion (Kim et al., 2012). Oxidative stress is intimately associated with neuroinflammation, and there has been found to be a vicious circle connecting oxidative stress and inflammation in AD (Rosales-Corral et al., 2010; Quintanilla et al., 2012; Joshi and Praticò, 2015). It has been observed that the redox status modulates inflammatory factors involvement in signaling processes, which are critical mediators of oxidative stress and inflammation, causing neurodegeneration (Mrak and Griffin, 2005; Kierdorf et al., 2010). Activation of glial cells and increased cytokine production is also induced by oxidative stress and, in turn, glial activation leads to the release of other neurotoxic factors such as ROS and nitric oxide (NO), which further exacerbate neuronal damage (Town et al., 2005; Block et al., 2007; Michelucci et al., 2009; von Bernhardi et al., 2010). Consequently, the resultant cellular damage amplifies the inflammatory response, with glial activation and leukocyte recruitment, leading to further inflammation in the AD brain (Figure 1).

## Inflammation in AD Pathogenesis

Besides oxidative stress damage, inflammatory responses are also closely associated with AD pathology. Inflammation occurs in pathologically vulnerable regions of the AD brain, with increased expression of acute-phase proteins and pro-inflammatory molecules, which create a chronic and self-sustaining inflammatory process, involving activated glia cells, and stressed neurons (Mrak and Griffin, 2005; Perry et al., 2010; Morales et al., 2014). Thus chronic inflammation plays a basic role in the progression of neuropathological changes in AD, resulting in neuronal dysfunction and death. The importance of neuroinflammation has emerged from several studies of AD brains, which have evidenced the activation and proliferation of glial cells, together with enhanced release of inflammatory mediators (cytokines, chemokines, growth factors, and other mediators) and free-radical-mediated oxidative stress (ROS, NO, and other radicals) (Glass et al., 2010; Heneka et al., 2010; McGeer and McGeer, 2010; Holmes and Butchart, 2011; Azizi and Mirshafiey, 2012; Rubio-Perez and Morillas-Ruiz, 2012; Lyman et al., 2014; Figueiredo-Pereira et al., 2015). Microglia are key players in the disease process, and once activated they present cell-surface antigens commonly present on monocytes and macrophages (Latta et al., 2014). Microglial activation thus leads to the initiation of an innate immune response, dominated by the release of pro-inflammatory cytokines (Town et al., 2005; Mrak, 2012; Weitz and Town, 2012). Incidental to this is also the phagocytosis of fibrils and large aggregates of Aβ, suggesting an initial neuroprotective defense mechanism (D’Andrea et al., 2004; Colton and Wilcock, 2010). Further, microglia can also secrete a number of soluble factors, such as glia-derived neurotrophic factor, which are potentially beneficial to the survival of neurons (Liu and Hong, 2003). Although the initial purpose of microglial activation is to counteract the detrimental effects induced by the pathological features, it subsequently leads to the release of high concentrations of neurotoxic factors, such as inflammatory molecules, NO, ROS, proteolitic enzymes, complementary factors, and excitatory amino acids, which further exacerbate cell damage (Michelucci et al., 2009). Moreover, in later stages of the disease, the overproduction of inflammatory cytokines makes the microglia phagocytically inactive, especially against insoluble oligomers and high concentrations of Aβ (Hickman et al., 2008; Krabbe et al., 2013).

A growing body of evidence also suggests that the central nervous system (CNS) and systemic inflammation cannot be viewed in isolation. Systemic inflammation might exarcerbate behavioral symptoms and accelerate AD progression by increasing the production of local pro-inflammatory cytokines and chemokines, as well as of ROS and NO (Holmes, 2013). The detrimental effects of peripheral inflammatory molecules in the brain of AD patients chiefly occur because these mediators can easily enter the brain, together with infiltrating leukocytes, thanks to increased permeability of the blood-brain barrier (BBB) as the disease progresses (Leoni et al., 2003; Popescu et al., 2009; Takeda et al., 2014). Attention is also now being paid to the participation of Toll-like receptors (TLRs) in inflammation and neurodegeneration. The recruitment of TLRs contributes to inflammation by amplifying the release of inflammatory molecules, thus playing an important role in the impact of inflammation on neuronal function and death (Drouin-Ouellet and Cicchetti, 2012).

However, it also remains unclear in the case of inflammation whether this process is a cause or a consequence of AD. Clinical and experimental studies support the appearance of neuroinflammatory changes already at the early stages of AD, even before the formation of extracellular Aβ deposits and intracellular NFT accumulation (Sheng et al., 2000). The release of pro-inflammatory cytokines and enzymes could affect the normal behavior of neuronal cells, leading to cell dysfunction and abnormalities such as Aβ peptides and NFT accumulation, events in the pathway leading to neuronal degeneration. Inflammatory molecules, and a number of stress conditions, enhance APP levels and the amyloidogenic processing of APP to induce Aβ_1–42_ peptide production. This, in turn, inhibits the formation of the soluble APP fraction that seems to have a neuronal protective effect (Fassbender et al., 2000; Misonou et al., 2000). Conversely, it has been demonstrated that intraneuronal Aβ and soluble Aβ oligomers activate microglia in the earliest stages of AD, even before senile plaque and NFT formation, in particular when cells are stressed (Ferretti and Cuello, 2011; Khandelwal et al., 2011). Moreover, Aβ and NFT have also been shown to trigger the release by activated glia cells of pro-inflammatory mediators, free radicals, and other neurotoxic substances, implying that the development of AD leads to the initiation of several self-propagating cycles (Vukic et al., 2009; Morales et al., 2013). Fibrillar Aβ can also activate microglia by binding to cell membranes via specific receptors, including a multi-receptor complex involving CD36, α_6_β_1_-integrin, and CD47 (Verdier et al., 2004; Yu and Ye, 2015), while the disruption of the *APP* gene and of its proteolytic products delays and decreases microglial activation (DeGiorgio et al., 2002). Once activated, microglia may also recruit and activate astrocytes, which actively enhance the inflammatory response to extracellular Aβ deposits (Jo et al., 2014). Aggregated amyloid fibrils and neurotoxic inflammatory molecules, secreted by glial cells, intensify neuronal dysfunction and death, either alone or in concert (Brown and Bal-Price, 2003; Findeis, 2007). In this context, activated glia cells and the released inflammatory molecules, together with other components of the immune response, are often present in proximity to neurons and areas of amyloid plaque (Abbas et al., 2002; Serrano-Pozo et al., 2013). Of note, microglia have also been suggested to be preferentially associated with certain types of amyloid deposits (D’Andrea et al., 2004). Furthermore, astrocytes are known to play a critical role in Aβ clearance, in providing trophic support to neurons, and in forming a protective barrier between Aβ deposits and neurons. The presence of large numbers of astrocytes associated with Aβ plaques suggests that these lesions induce the release of chemotactic molecules that mediate astrocyte recruitment. However, it has been suggested that astrocytes could also be a source for Aβ peptides, because they overexpress BACE1 in response to chronic stress (Roßner et al., 2005; Wang et al., 2011). While Aβ has been widely demonstrated to be pro-inflammatory, the association between microglial activation and tau pathology development is still not supported by direct evidence, since neurofibrillary disorder occurs both in the presence and absence of neuroinflammation (Streit et al., 2014), and intraneuronal NFT lesions usually precede the formation of Aβ aggregates (Braak and Del Tredici, 2004).

Although it has been reported that microglia and astrocytes actively promote disease development, and play pivotal roles in amyloid deposition (Wegiel et al., 2004), conversely it has also been reported that microglia are protective and may remove amyloid deposits, thus having no effect on AD development (Simard et al., 2006; Grathwohl et al., 2009). Moreover, and interestingly, it has been suggested that infiltrating macrophages from the circulation, rather than microglia, play a central role in clearing Aβ deposits in cerebral amyloid angiopathy (Hawkes and McLaurin, 2009), but that these cells can also play a determinant role in AD development (Gate et al., 2010; Rezai-Zadeh et al., 2011).

Of note, while neurons were traditionally believed to be passive bystanders in neuroinflammation, some recent evidence suggests that not only astrocytes and microglia, but also neurons themselves, contribute to the chronic inflammation in AD, by serving as a source of inflammatory molecules (Tchelingerian et al., 1994; Yan et al., 1995; Murphy et al., 1999; Suzuki et al., 1999; Acarin et al., 2000; Heneka and O’Banion, 2007; Rubio-Perez and Morillas-Ruiz, 2012; Ramesh et al., 2013). An increase in pro-inflammatory molecule expression has also been observed in human neuroblastoma SH-SY5Y cells after incubation with some cholesterol oxidation products (known as oxysterols) potentially implicated in AD pathogenesis (Testa et al., 2014).

## Brain Cholesterol Metabolism and AD

The human brain contains approximately 25% of the body’s cholesterol; this is essential for its normal functioning, being a major component of neuronal cell membranes and an essential factor in membrane fluidity. In the adult brain, cholesterol is mostly present in its non-esterified form; however, small amounts of desmosterol and cholesteryl ester are also present. The brain is separated from the peripheral circulation by the BBB, which prevents the dietary intake of cholesterol being transported from the circulation to the brain, since lipoproteins do not cross the BBB. This means that nearly all the brain cholesterol is synthesized *de novo* within the CNS, from 3-hydroxy-3-methyl-glutaryl-coenzyme A reductase, through a complex series of reactions involving more than 20 enzymes; cholesterol metabolism is, thus, regulated independently of that in the peripheral tissues (Vance et al., 2005; Dietschy, 2009). Because neurons do not efficiently synthesize cholesterol, they rely on astrocytes as an external source. Astrocytes meet neuronal cholesterol demands by secreting ApoE-cholesterol complexes, which are transported to the neurons for their development and function. On note, ApoE transcription is regulated by 24-hydroxycholesterol (24-OH) released by the neurons via the liver X receptor (LXR), this cholesterol oxidation product being one of its natural ligands (Pfrieger, 2003; Nieweg et al., 2009). LXR is a nuclear receptor that regulates the expression of specific genes involved in cholesterol efflux and metabolism, such as ATP-binding cassette transporters A1 and G1 (*ABCA1* and *ABCG1*), and *ApoE* (Sodhi and Singh, 2013).

ApoE is the brain’s principal cholesterol-carrier protein; through its receptors it regulates the redistribution and homeostasis of cholesterol within the brain. Humans express three naturally-occurring alleles of the *ApoE* gene: ε2, ε3, and ε4. Murine studies have shown that astrocytes and microglia are the primary ApoE secreting cells in the brain, but neurons can express ApoE under conditions of excitotoxic injury (Xu et al., 2006).

The association between ApoE polymorphism and AD is presumably related to disorders in cholesterol transport. In this connection, it has been found that receptors recognizing ApoE are widely expressed in the brain of AD patients. Of note, AD is accelerated in ApoEε4 individuals: those who are homozygous for the ε4 allelic variant of ApoE have a 50–90% higher chance of developing AD by age 85 years than those carrying ε2 and ε3, showing that this ApoE isoform is one of the major risk factors for AD (Puglielli et al., 2003; Evans et al., 2004; Bu, 2009; Kim et al., 2009a; Martins et al., 2009; Schipper, 2011). However, the mechanisms whereby ApoE isoforms affect the risk of AD are still obscure. One hypothesis is that ApoEε4 protein accelerates the uptake of cholesterol-rich particles by the vasculature, leading to more rapid disease progression: compared to ApoEε4, ApoEε2 and ApoEε3 proteins show reduced receptor binding.

Although ApoE facilitates intracellular Aβ degradation by microglia, reducing intracellular cholesterol levels (Lee et al., 2011) by accelerating its clearance by binding Aβ and forming a stable complex (Sagare et al., 2007; Jiang et al., 2008), ApoE is, conversely, essential for Aβ aggregation and deposition, promoting Aβ fibrillization and plaque formation, as well as tau hyperphosphorylation and NFT formation (Cam and Bu, 2006; Bu, 2009; Marzolo and Bu, 2009). Of note, in the AD-affected brain, ApoE colocalizes with cholesterol and fibrillar Aβ in senile plaques (Burns et al., 2003b). It has also been shown that the *ApoEε4* genotype enhances Aβ production and Aβ fibril formation *in vitro*, as well as in transgenic mice with mutated APP, more than in mice expressing ApoEε3 (Holtzman et al., 2000; Carter et al., 2001; Ye et al., 2005). In addition, ApoEε4 synergizes with Aβ toxicity (Ji et al., 2002). Conversely, a striking reduction in amyloid deposits has been observed in all brain regions of ApoE-null mice (Bales et al., 1999). It has also been suggested that, in AD patients with ApoEε4, a decreased ability to clear Aβ contributes to increasing Aβ accumulation and amyloid plaque formation (Deane et al., 2008). In addition, ApoE may mediate Aβ cell internalization, by binding to the low density lipoprotein receptor-related protein (LRP; Herz and Beffert, 2000). Further, like APP, ApoE also undergoes cleavage, and ApoEε4 is more susceptible to cleavage than is ApoEε3. Fragments of ApoE, such as Aβ, can be toxic, causing AD-like neurotoxicity in mouse models (Harris et al., 2003; Brecht et al., 2004); the lipid-binding region of ApoE is required for this toxicity (Chang et al., 2005).

Accumulating evidence also supports a key role for human ApoE in modulating neuroinflammation (Maezawa et al., 2006c; Keene et al., 2011; Tai et al., 2015). Many studies have reported that ApoEε4 induces a detrimental neuroinflammatory phenotype, both in peripheral and CNS inflammation. *In vitro* data, using microglia isolated from ApoE transgenic mice, has demonstrated *ApoE* genotype-specific modulation of TLR4/lipopolisaccaride (LPS) induced inflammation. In particular, LPS-induced pro-inflammatory cytokines are more strongly expressed in ApoEε4 and ApoEε3 (Maezawa et al., 2006b). Moreover, in microglia treated with LPS/interferon γ, pro-inflammatory cytokine levels were higher, and anti-inflammatory cytokine levels were lower, in ApoEε4 compared to ApoEε3. Unlike what occurs in microglia, in astrocytes TLR4/LPS-induced secretion of pro-inflammatory cytokines follows the pattern: ApoEε2 > ApoEε3 > ApoEε4 via differential nuclear factor-κB (NF-κB) activation (Maezawa et al., 2006a). With regard to ApoE modulation of Aβ-induced neuroinflammation, the available results are scarse. It has been demonstrated that impaired ApoEε4 function modulates the effects induced by Aβ on inflammatory receptor signaling, by amplifying the detrimental pathway TLR4-p38α and suppressing the beneficial pathway interleukin-4R-nuclear receptor (Tai et al., 2015). Importantly, ApoE also modulates BBB function, by a process that may be considered neuroinflammatory. It has been observed that a lack of murine ApoE combined with the expression of ApoEε4, but not of ApoEε2 nor ApoEε3, leads to BBB breakdown by activating the pro-inflammatory pathway cyclophilin A-NF-κB-matrix metalloproteinase 9 in pericytes. Consequently, blood-derived neurotoxic proteins are taken up by neurons, and microvascular and cerebral blood flows are reduced. It has been shown that the vascular defects in ApoE-deficient and ApoEε4-expressing mice precede neuronal dysfunction and can initiate neurodegenerative changes (Bell et al., 2012).

In addition to ApoE, other LXR-responsive genes involved in cholesterol efflux are the *ABCA1* and *ABCG1* (Voloshyna and Reiss, 2011). ABCA1 is reported to be involved in ApoE metabolism and Aβ production, as well as in the modulation of amyloid plaque formation in the CNS (Koldamova et al., 2010; Kim et al., 2011). A close correlation between Aβ and ABCA1 levels has been demonstrated, since, in cultured astrocytes, Aβ inhibits ABCA1 expression (Canepa et al., 2011). In addition, it has also been shown that AD transgenic mice lacking ABCA1 develop increased Aβ levels and senile plaques, in the absence of changes in APP processing (Wahrle et al., 2004). By contrast, in transgenic mice overexpressing ABCA1, the increased ABCA1 function significantly decreases Aβ deposition (Wahrle et al., 2008). Another ABC transporter, ABCA7, has been found to stimulate cellular cholesterol efflux to ApoE-containing particles in the same way as ABCA1 (Chan et al., 2008).

Cholesterol transport and homeostasis are thus closely linked to multiple aspects of Aβ biology, since cholesterol levels influence the production and deposition of the pathogenic Aβ peptides (Burns et al., 2003a; Hughes et al., 2014; Lane-Donovan et al., 2014). Cholesterol has indeed been shown to directly modulate the processing of APP in neuronal cell cultures (Ehehalt et al., 2003), probably by promoting β-secretase activity (Xiong et al., 2008). However, the mechanisms whereby cholesterol affects Aβ production and deposition are still not fully understood. A change in membrane properties and distribution of cholesterol has been suggested as a possible mechanism (Shobab et al., 2005). Cholesterol is mainly concentrated in membrane microdomains termed lipid rafts, which are considered to be the site of the amyloidogenic pathway (Cordy et al., 2006; Vetrivel and Thinakaran, 2010). Further, it has been shown that cellular cholesterol, especially when levels in the membrane are elevated, binds directly to APP at its C terminal transmembrane domain (Harris, 2008; Beel et al., 2010); as a consequence of the binding APP is inserted into the phospholipid monolayers of the lipid rafts and other organelles, where β- and γ-secretases reside (Wahrle et al., 2002; Beel et al., 2010). The amyloidogenic pathway of APP processing is thus linked to cholesterol levels in these microdomains: β- and γ-secretase activities are stimulated by high, and inhibited by low levels of cholesterol (Grimm et al., 2008; Xiong et al., 2008). Conversely, in the non-amyloidogenic pathway, APP is processed by α-secretase in non-raft domains, and this event is promoted by a decreased cellular cholesterol level (Reid et al., 2007). Moreover, α-secretase can be forced to associate with lipid rafts thus inactivating the amyloidogenic pathway (Harris et al., 2009). Of note, it has also been suggested that high levels of non-esterified cholesterol alone might not affect APP processing, while the conversion of free cholesterol to esterified cholesterol might up-regulate APP processing and Aβ generation (Puglielli et al., 2001). Conversely, inhibition of the enzyme acyl-coenzyme A:cholesterol acyl-transferase 1, which esterifies cholesterol, leads to the formation of smaller amounts of cholesteryl esters and Aβ (Bhattacharyya and Kovacs, 2010). These observations suggest that the balance between non-esterified and esterified cholesterol is a fundamental point controlling the amyloidogenic pathway.

It has also been shown that increased cholesterol levels in the lipid bilayers favor Aβ binding to cell membranes (Kakio et al., 2001). Additionally, cholesterol interacts with the peptide as soon as it inserts into the lipid bilayer, and accelerates its recruitment and oligomerization (Fantini and Yahi, 2010). Indeed, cholesterol enhances Aβ to form neurotoxic aggregates (Yanagisawa, 2005), and binds avidly to Aβ protofibrils at level of lipid rafts where fibrillogenesis of these peptides has been proposed to take place (Kakio et al., 2002; Harris, 2008). Conversely, other research has demonstrated that cholesterol decreases the Aβ-induced changes in structure and morphology of lipid rafts, hindering β-sheet formation in membranes, and thereby reducing peptide insertion, aggregation, and cytotoxicity (Arispe and Doh, 2002; Curtain et al., 2003; Qiu et al., 2011).

The effect of Aβ on cholesterol metabolism has also been investigated: Aβ, especially the oligomeric rather than the monomeric form, may alter the intracellular trafficking and homeostasis of cholesterol, by promoting the release from the cells of cholesterol and other lipids, in the form of Aβ-lipid complexes (Michikawa et al., 2001). It has also been reported that Aβ fibrils down-regulate cholesterol metabolism in cultured neurons (Gong et al., 2002). Additionally, the intracellular domain of APP, which is released upon γ-secretase cleavage of APP, may act as a transcriptional suppressor of LRP1, leading to the down-regulation of cellular cholesterol uptake (Liu et al., 2007) and synaptic failure (Koudinov and Koudinova, 2005), as well as enhancement of tau phosphorylation (Fan et al., 2001). It has also been shown that extracellular cholesterol accumulates in the senile plaques of AD patients, and in transgenic mice expressing the Swedish Alzheimer mutation APP751, by binding to aggregated Aβ (Mori et al., 2001).

Given the above considerations, it appears that cholesterol distribution and trafficking within brain cells, rather than the total amount of cholesterol in the neurons, play key roles in APP processing and in the amyloid cascade during AD progression. Conversely, Aβ may affect cellular cholesterol dynamics, such as transport, distribution, and binding, which in turn have a variety of effects on AD-related pathologic changes leading to neurodegeneration.

A number of epidemiological studies also suggest a positive correlation between hypercholesterolemia and susceptibility to AD, in particular in individuals with the *ApoEε4* genotype, which influences cholesterol metabolism, although this relationship is still the subject of considerable controversy (Shobab et al., 2005; Panza et al., 2006; Jenner et al., 2010; Wood et al., 2014; Luckhoff et al., 2015).

Elevated dietary cholesterol has been reported to increase senile plaque formation in numerous animal models. In double transgenic (APP/PS) mice, consumption of a high-cholesterol diet (7 weeks) elevated Aβ deposition in the CNS (Refolo et al., 2000). In addition, in transgenic mice, a typical Western diet (1% cholesterol), increased Aβ accumulation and plaque burden, particularly in the dentate gyrus of the hippocampus (Hooijmans et al., 2007). APP23 mice fed a high-fat/high-cholesterol diet for 4 months showed the AD phenotype. This resulted in significantly worse memory deficits than in the same mice fed with a normal diet (Fitz et al., 2010). Furthermore, studies using New Zealand white rabbits have demonstrated that a diet inducing hypercholesterolemia doubles the Aβ concentration in the hippocampal cortex (Sparks et al., 2000). In addition to increasing Aβ, cholesterol-enriched diets increased tau phosphorylation and oxidative stress in rabbit brains (Ghribi et al., 2006; Jaya Prasanthi et al., 2008). It has also been shown that cholesterol colocalizes with fibrillar Aβ in the amyloid plaques of transgenic mice (Burns et al., 2003b). Conversely, a study on guinea pigs showed that lowering total cholesterol profile by administering statins decreased cerebral Aβ production and accumulation (Fassbender et al., 2001).

Although systemic lipoprotein carrying cholesterol cannot cross the BBB, oxysterols, formed during oxidative stress, can cross the BBB, partly because they may have damaging effects on the BBB’s integrity and function (Dias et al., 2014). This consideration supports the idea that oxysterols are the link between hypercholesterolemia and AD.

## The Involvement of Oxysterols in AD Pathogenesis

**Figure 2:**
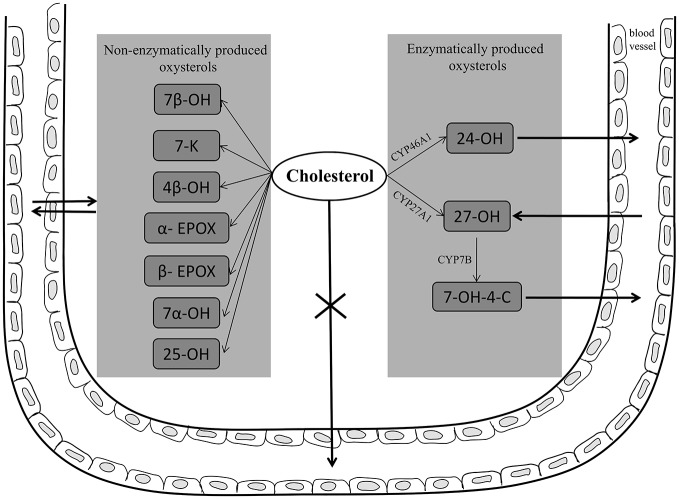
**Enzymatically and non-enzymatically produced oxysterols in AD brain and their fluxes across the blood brain barrier (BBB)**. In neuronal cells, cholesterol is converted into 24-hydroxycholesterol (24-OH) by the enzyme CYP46A1; 24-OH, unlike cholesterol, diffuses across the BBB into the systemic circulation. To a lesser extent, cholesterol is also converted into 27-hydroxycholesterol (27-OH) by the enzyme CYP27A1, and then into 7α-hydroxy-3-oxo-4-cholestenoic acid (7-OH-4-C) by the enzyme CYP7B; crossing the BBB, 7-OH-4-C reaches the liver where it is eliminated. However, most 27-OH flows from the circulation into the brain, since it can cross the BBB. In addition, other oxysterols, such as 7β-hydroxycholesterol (7β-OH), 7-ketocholesterol (7-K), 7α-hydroxycholesterol (7α-OH), 4β-hydroxycholestrerol (4β-OH), 5α, 6α- and 5β, 6β-epoxicholesterol (α- and β-EPOX), and 25-hydroxycholesterol (25-OH), have been found in AD brain deriving from brain cholesterol autoxidation. Potentially these oxysterols, as well as other cholesterol metabolites, can cross the BBB.

To maintain cholesterol homeostasis in the brain, and because the brain cannot degrade cholesterol, there must be a mechanism to eliminate excess cholesterol, transporting it through the BBB into the systemic circulation, thus preventing its accumulation. The most important such mechanism operates via the conversion of cholesterol to oxysterols. Cholesterol is converted into the relatively polar oxysterol 24-OH, which is produced in the brain almost exclusively by cholesterol 24-hydroxylase (CYP46A1) expressed in neurons, and which, unlike cholesterol itself, diffuses across the BBB into the systemic circulation. To a lesser extent, cholesterol is also converted into 27-OH in the brain by cholesterol 27-hydroxylase (CYP27A1), and then into 7α-hydroxy-3-oxo-4-cholestenoic acid (7-OH-4-C) by the enzyme CYP7B; crossing the BBB, 7-OH-4-C reaches the liver where it is eliminated (Björkhem, 2006; Meaney et al., 2007; Björkhem et al., 2009). However, most 27-OH has been found to flow from the systemic circulation into the brain, since it can cross the BBB; here it acts as an important link between extracerebral and intracerebral pools of cholesterol, and may contribute to the negative effects of hypercholesterolemia in the brain (Heverin et al., 2005; Björkhem, 2006; Sharma et al., 2008). A further oxysterol, 7β-hydroxycholesterol (7β-OH), derives from cholesterol oxidation in the brain, following its interaction with APP and Aβ (Nelson and Alkon, 2005). In addition to those oxysterols, others including 7α-hydroxycholesterol (7α-OH), 4β-hydroxycholestrerol (4β-OH), 5α, 6α- and 5β, 6β-epoxicholesterol (α- and β-EPOX) and 7-ketocholesterol (7-K), have recently been identified post-mortem in human AD brains, and their concentrations compared across the disease states (Hascalovici et al., 2009). Of note, the study authors observed that the levels of potentially amyloidogenic sterol species derived from the auto-oxidation of cholesterol, like β-EPOX, were higher at the mild cognitive impairment disease stage. Oxysterols have also been identified in mouse brains and, in addition to the above cited cholesterol derivatives, consistent levels of 25-hydroxycholesterol (25-OH) were found (Ahonen et al., 2014). Increased levels of 24-OH, 7-K, and β-EPOX have also been detected in areas of the rat hippocampus undergoing gliosis and neuroinflammation, after excitotoxic injury (He et al., 2006; Kim et al., 2010). Further, because oxysterols can cross the BBB, the flux of more than 20 cholesterol metabolites between brain and circulation has very recently been verified in 20 patients. Differences in concentrations, between jugular and forearm veins, of 18 oxysterols, 5 cholestenoic acids and 3 cholenoic acids were measured. The study reported that 24-OH and 7-OH-4-C, of enzymatic origin, but also 6-oxo-5α-hydroxycholesterol, 7β-OH, 7-K, which are formed from cholesterol by ROS, are exported from the brain, while 27-OH is imported into brain (Iuliano et al., 2015). Of these cholesterol metabolites, that exported in the largest quantities is 24-OH. Two other cholesterol metabolites, 7α, 25-dihydroxycholest-4-en-3-one and 7α, (25R)26-hydroxycholest-4-en-3-one, were reported to be exported from the brain (Crick et al., 2015b). Oxysterols and cholestenoic acids have also been identified and quantified in mouse cerebrospinal fluid (CSF), and the findings compared with concentrations of the same metabolites found in the plasma, in order to clarify cholesterol metabolism. Concentrations of oxysterols were lower in the CSF than in the plasma, but 7α, 24-dihydroxycholesterol and 7α, 24-dihydroxycholest-4-en-3-one, both of enzymatic origin, were only identified in the CSF (Crick et al., 2015a). These data clearly demonstrate that there are several routes by which cholesterol metabolites may be exported or imported from the brain (Figure 2).

The idea has therefore gained ground that, owing to their ability, unlike cholesterol, to cross the BBB, oxysterols might be the missing link between altered brain cholesterol metabolism and AD pathogenesis, as well as between hypercholesterolemia and AD (Gamba et al., 2012). Although this means that the brain can eliminate excess amounts of oxysterols, it could conversely allow toxic amounts of these compounds, present in the bloodstream, to accumulate in the brain, as in the case of 27-OH. The key role of oxysterols in AD pathogenesis has been strongly supported by the last decade’s research, pointing to the involvement of these oxysterols in the amyloid cascade.

To date, although contradictory results could obviously arise from future findings on the other cholesterol metabolites found in the brain, the oxysterols most widely considered to be potentially implicated in the pathogenesis of AD are 24-OH and 27-OH, both of enzymatic origin (Iuliano, 2011; Jeitner et al., 2011; Leoni and Caccia, 2011; Gamba et al., 2012; Hughes et al., 2013; Marwarha and Ghribi, 2014; Noguchi et al., 2014; Table 1).

**Table 1:** **24-hydroxycholesterol and 27-hydroxycholesterol levels in Alzheimer’s disease patients compared with healthy controls**.

Oxysterol	Levels of oxysterol in AD subjects compared with control subjects	Reference
**24-hydroxycholesterol**	↓ Brain levels	Heverin et al. (2004)
	↑ CSF levels	Papassotiropoulos et al. (2002), Schönknecht et al. (2002) and Leoni et al. (2004)
	↓ Plasma levels	Bretillon et al. (2000) and Kölsch et al. (2004)
	↑ Plasma levels	Lütjohann et al. (2000)
	= Plasma levels	Iuliano et al. (2010)
**27-hydroxycholesterol**	↑ Brain levels	Heverin et al. (2004) and Shafaati et al. (2011)
	↑ CSF levels	Leoni et al. (2004)
	↓ Plasma levels	Kölsch et al. (2004)
	= Plasma levels	Iuliano et al. (2010)

*CSF, cerebrospinal fluid*.

Higher levels of 24-OH than in unaffected individuals have been found in the peripheral circulation and CSF of early-stage AD patients, suggesting that cholesterol turnover in the brain increases during the neurodegenerative changes of AD (Lütjohann et al., 2000; Papassotiropoulos et al., 2002; Kölsch et al., 2004). Conversely, plasma levels of 24-OH were lower in patients with later stages of AD than in unaffected individuals, suggesting that the rate of cholesterol transport slows as the disease progresses (Bretillon et al., 2000; Kölsch et al., 2004). These apparently contradictory results may be rationalized by considering that increased plasma levels of 24-OH reflect ongoing neurodegeneration and/or demyelinization, whereas decreased plasma levels in later stages reflect a selective loss of neuronal cells expressing the enzyme CYP46A1 (Björkhem and Meaney, 2004). The decrease of 24-OH in the AD brain may also be the result of an increase in total free cholesterol (likely derived from cell membrane collapse and widespread myelin release) that exceeds the brain’s capacity to convert it to 24-OH (Vaya and Schipper, 2007). However, it has been observed that, in glial cells, and especially around senile plaques, there is an ectopic induction of CYP46A1, leading to some 24-OH production, but without compensating for the decrease of that oxysterol (Bogdanovic et al., 2001; Brown et al., 2004). Another study, however, has found that plasma levels of 24-OH and 27-OH in AD patients are not significantly different from control values (Iuliano et al., 2010). A small fraction of total 24-OH excretion occurs via the CSF, and the 24-OH concentration is increased in the CSF of AD patients, probably reflecting neuronal damage and loss rather then metabolically active neuronal cells (Schönknecht et al., 2002; Leoni et al., 2004).

It has also been reported that, in critical areas of post-mortem AD brains, as well as in aged mice expressing the Swedish Alzheimer mutation APP751, 24-OH levels are decreased and those of 27-OH increased (Heverin et al., 2004). Increased levels of 27-OH were also found in the brains of patients carrying the Swedish APP670/671 mutation (Shafaati et al., 2011). As a consequence of neuron loss, expression of CYP27A1 may also be reduced; however, 27-OH levels remain elevated because CYP27A1 is also expressed in astrocytes and oligodendrocytes, leading to *in situ* generation of 27-OH (Brown et al., 2004). Accumulation of 27-OH in the brain is also due to the increased flux of this oxysterol across the BBB, because of either hypercholesterolemia associated to oxidative stress (Björkhem, 2006), or damaged BBB integrity (Leoni et al., 2003). There is a positive correlation between levels of cholesterol and those of 27-OH in the circulation, and the high flux of 27-OH from the peripheral circulation into the brain suggests that this oxysterol may be the link between hypercholesterolemia and AD. An alternative explanation for the accumulation of 27-OH is reduced activity of CYP7B, the neuronal enzyme responsible for 27-OH metabolism; this reduction arises from the reduced CYP7B expression in the brain of AD patients, because of neuron loss (Yau et al., 2003). Moreover, high CSF levels of 27-OH were found in mild cognitive impairment and AD patients (Leoni et al., 2004).

From these considerations, it is clear that the balance between 24-OH and 27-OH levels is important. Oxysterol homeostasis in the brain is tightly regulated, specific levels being maintained in various brain regions. For example, the 27-OH:24-OH ratio is ~1:8 in the frontal cortex, 1:5 in the occipital cortex, and 1:10 in the basal ganglia, and the increased ratio of 27-OH to 24-OH in AD brains is consistent with AD pathogenesis. Thus it is likely that reduced levels of 24-OH may accelerate disease progression, and that the increased levels of 27-OH may be insufficient to compensate for this: the shift in balance between the two oxysterols might lead to increased generation and accumulation of Aβ with consequent neurodegeneration (Heverin et al., 2004; Björkhem, 2006; Björkhem et al., 2009). In a retrospective study on cardiovascular patients with evidence of cerebrovascular disease, higher plasma levels of 24-OH and a higher 24-OH/27-OH ratio were found to be associated with the development of incidental cognitive impairment over 8 years of follow-up (Hughes et al., 2012). However, opinions still differ about the involvement of 24-OH and 27-OH in APP processing and Aβ production.

To date, the conversion of cholesterol into 24-OH, by inducing CYP46A1 activity, has been considered to exert a protective action on the brain, mainly by regulating cholesterol homeostasis and favoring the efflux of its excess from the brain to the blood, but also by preventing Aβ generation (Björkhem et al., 2009). Astrocytes are sensitive to levels of 24-OH, which regulates the expression of LXR-responsive genes involved in cholesterol homeostasis (i.e., *ABCA1*, *ABCG1* and *ApoE*) (Abildayeva et al., 2006). Indeed, 24-OH acts as an endogenous ligand of LXR. Conversely, it has been reported that brain accumulation of 27-OH antagonizes the preventive effect of 24-OH on generation of Aβ and that it is potentially toxic (Shafaati et al., 2011). Since the flux of 27-OH across the BBB increases under conditions of hypercholesterolemia (Björkhem, 2006), or in the case of increased BBB permeability (Leoni et al., 2003), the inhibitory effect of 24-OH on Aβ generation would consequently be reduced.

Studies on human neuroblastoma cells and on brain tissues have somewhat clarified the different effects of 24-OH and 27-OH on APP levels and processing: 24-OH may favor the non-amyloidogenic pathway, with consequent inhibition of Aβ formation, whereas 27-OH is thought to stimulate the amyloidogenic pathway, with production of Aβ as well as tau hyperphosphorylation (Bu, 2009; Prasanthi et al., 2009; Marwarha et al., 2010). However, the mechanisms underlying their different effects are still unclear.

In human SH-SY5Y neuroblastoma cells, 24-OH appears to exert a unique modulatory effect on APP processing: it directly increases α-secretase activity, as well as elevating the α/β-secretase activity ratio; conversely, 27-OH enhances the generation of Aβ (Famer et al., 2007). *In vitro* experiments suggest that 24-OH reduces Aβ production, by down-regulating APP trafficking via enhancement of the complex formation of APP, also up-regulating glucose-regulated protein 78 in the endoplasmic reticulum. The inhibitory effect of 24-OH was reduced in glucose-regulated protein 78 knockdowned cells (Urano et al., 2013). In rat primary cortical neurons, both 24-OH and 27-OH were found to be inhibitors of Aβ secretion, 24-OH being approximately 1000 times more potent than 27-OH (Brown et al., 2004). SH-SY5Y cells incubated with 27-OH release higher levels of Aβ_1–42_ and APP as well as of BACE1. Conversely, cells incubated with 24-OH do not release increased Aβ_1–42_ levels, and are associated with increased levels of sAPPα, suggesting that 24-OH favors APP processing via the non-amyloidogenic pathway (Prasanthi et al., 2009). Another study, on hippocampal slices from adult rabbits, found that 27-OH increases Aβ accumulation by reducing levels of insulin-like growth factor 1, a neurotrophic factor that promotes neurogenesis and has a neuroprotective effect (Sharma et al., 2008). This oxysterol has also been found to increase both BACE1 and Aβ levels in retinal pigment epithelial cells (Dasari et al., 2010). In neuronal SK-N-BE cells, 24-OH and 27-OH have both been shown to enhance expression and activity of the β-secretase of the amyloidogenic pathway of APP processing, leading to increased Aβ generation and accumulation in those cells (Gamba et al., 2014; Table 2).

**Table 2:** **Effects of 24-hydroxycholesterol and 27-hydroxycholesterol on the amyloidogenic pathway**.

Oxysterol	Dosage	Effects	Experimental model	Reference
**24-hydroxycholesterol**	*C* = 10 μM	↓ Aβ production	Primary culture of rat cortical neurons	Brown et al. (2004)
	*C* = 5 μM	↑ α-secretase activity ↓ β-secretase activity	Undifferentiated human neuroblastoma cell line SH-SY5Y	Famer et al. (2007)
	*C* = 10 μM	↑ α-secretase activity no effect on APP, BACE1 and Aβ level	Undifferentiated human neuroblastoma cell line SH-SY5Y	Prasanthi et al. (2009)
	*C* = 1–10 μM	↑ APP level ↓ Aβ production no effect on β-secretase activity	Undifferentiated SH-SY5Y cells and Chinese hamster ovary (CHO) cells	Urano et al. (2013)
	*C* = 1 μM	↑ APP level and Aβ production ↑ α-secretase level ↑ β-secretase level and activity	Differentiated human neuroblastoma cell line SK-N-BE	Gamba et al. (2014)
**27-hydroxycholesterol**	*C* = 1–15 μM	↓ Aβ production	Primary culture of rat cortical neurons	Brown et al. (2004)
	*C* = 5 μM	No effect on α- and β-secretase activity	Undifferentiated human neuroblastoma cell line SH-SY5Y	Famer et al. (2007)
	*C* = 10 μM	↑ Aβ production ↑APP and BACE1 level	Undifferentiated human neuroblastoma cell line SH-SY5Y	Prasanthi et al. (2009)
	*C* = 10–25 μM	↑ Aβ production ↑ BACE1 level	Retinal pigmented epithelial cells ARPE-19	Dasari et al. (2010)
	*C* = 5 μM	↑ Aβ production ↑ BACE1 level and activity	Undifferentiated human neuroblastoma cell line SH-SY5Y	Marwarha et al. (2013)
	*C* = 1 μM	↑ APP level and Aβ production ↑ α-secretase level ↑ β- and γ-secretase level and activity	Differentiated human neuroblastoma cell line SK-N-BE	Gamba et al. (2014)

*Aβ, amyloid β; APP, amyloid precursor protein; BACE1, beta-site amyloid precursor protein cleaving enzyme 1*.

24-OH has also been reported to be neurotoxic, but this effect may depend on its concentration. It has been demonstrated that high concentrations of 24-OH (25–50 μM) caused cell death when added to undifferentiated or differentiated SH-SY5Y cells: the effect was mediated by increased generation of free radicals (Kölsch et al., 2001). High concentrations of 24-OH (50 μM) also induce necroptosis, a form of programmed necrosis in neuronal SH-SY5Y cells (Yamanaka et al., 2011). In contrast, pretreatment of human neuroblastoma SH-SY5Y cells with sub-lethal concentrations of 24-OH induces adaptive responses, and protects the cells against subsequent cytotoxic stress induced by 7-K treatment, via transcriptional activation of the LXR signaling pathway. The cytoprotective effects of 24-OH disappeared in LXRβ-knockdowned cells, suggesting that this nuclear receptor may play a key role in the 24-OH-induced adaptive response. Adaptive responses are also induced by other oxysterols, such as 25-OH and 27-OH, both ligands of LXR, similarly to 24-OH (Okabe et al., 2013). However, in our recent studies, a very low concentration (1 μM) of 24-OH was found to markedly potentiate both the apoptotic and the necrogenic effects exerted by the Aβ_1–42_ peptide, on two human differentiated neuronal cell lines (SK-N-BE and NT-2) (Gamba et al., 2011), but also on human dental pulp progenitor cells differentiated into neuron-like cells (Testa et al., 2012). 24-OH appeared to interact with Aβ_1–42_ by strongly increasing intracellular ROS steady-state levels, an action not exerted by either 27-OH or 7β-OH (Gamba et al., 2011). This evidence supports the opinion that Aβ must form oligomers in order to induce neurotoxicity, and that the latter process is probably enhanced by redox imbalance. Additionally, 50 μM 24-OH has been shown to enhance the neurotoxic effect of the Aβ_1–42_ peptide in the human differentiated neuroblastoma cell line MSN, as well as augmenting ROS generation (Ferrera et al., 2008). Although in many of the various *in vitro* tests performed in our laboratory, 27-OH (1 μM) did not display neurotoxicity, in terms of necrosis and apoptosis (Gamba et al., 2011), conversely, the toxicity of 27-OH (5–20 μM) has been demonstrated in astrocyte cells (C6 cells). This oxysterol increased ROS levels and decreased antioxidant defense system levels, with consequent decrease of cell viability. In addition, 27-OH down-regulated the expression of the nuclear factor E2-related factor 2 signaling pathway (Ma et al., 2015).

It is known that hypertension is a risk factor for AD, and that angiotensin converting enzyme activity is increased in AD brains. In this connection, it has been suggested that 27-OH might up-regulate the renin-angiotensin system in AD brains. A positive correlation between angiotensin converting enzyme activity in the CSF, and both plasma and CSF levels of 27-OH, has been shown, as well as an increased production of angiotensinogen in rat primary neurons, astrocytes, and human neuroblastoma cells treated with 27-OH (Mateos et al., 2011).

Moreover, 7β-OH has been found to be neurotoxic at very low concentrations on cultured rat hippocampal neuronal cells, and may therefore contribute to neurodegeneration in AD brains. In the same study, it has also been shown that Aβ can oxidize cholesterol to form 7β-OH in a highly efficient mechanism and more actively than APP. Oxidation of cholesterol was accompanied by hydrogen peroxide production, suggesting that Aβ could contribute to the oxidative damage observed in AD (Nelson and Alkon, 2005). 7β-OH causes re-arrangement of the liquid-ordered phase which results in the formation of lipid rafts (Wang et al., 2008; Mitomo et al., 2009); it is also a potent inhibitor of α protein kinase C, an enzyme critical for memory consolidation and synaptic plasticity that is implicated in AD (Nelson and Alkon, 2005). Another oxysterol that might derive from the autoxidation of cellular cholesterol released during neurodegeneration is 7α-OH, which appeared to be responsible for SH-SY5Y cell death (Kölsch et al., 2000); a further possibility is 7-K. 7-K has been shown to enhance mitochondrial dysfunction in the neuronal PC12 cell line, leading to cell death (Kim et al., 2006; Kim and Lee, 2010; Jang and Lee, 2011). It has also been reported that incorporation of 7-K to lipid raft domains of plasma membranes triggers apoptotic signaling; α-tochopherol (vitamin E) reduces the cytotoxicity of 7-K by inhibiting its distribution to the lipid raft domains (Berthier et al., 2004; Royer et al., 2009). It has recently been suggested that 25-OH is an important regulator of cholesterol metabolism, as well as of humoral immunity (Diczfalusy, 2013; Waltl et al., 2013). Of note, it has also been hypothesized that Aβ deposition is not a central event in AD, but rather is subservient to 25-OH. Confirming this hypothesis, it has been observed that, in a large cohort of AD patients, specific *25-hydroxylase* haplotypes were associated with a complete absence of Aβ deposits in the brain, despite all other aspects of AD pathology being present (Papassotiropoulos et al., 2005). This suggests that neuroinflammation and 25-hydroxylase activation precede Aβ generation in the sequence of events leading to the disease. An interesting study on different immortalized, tumoral and normal cells of the CNS has found that oxysterols oxidized at C4, such as 4α-OH and 4β-OH, have no effect on cell viability and almost no effect on cell growth; conversely, oxysterols oxidized at C7, such as 7-K, 7α-OH, and 7β-OH, inhibit cell growth and decrease viability through their cytotoxic activity. These data suggest that 4α-OH and 4β-OH, the only oxysterols identified as having cytostatic properties, may be of some interest for attempts to counteract cell proliferation (Nury et al., 2013).

Oxysterols have also been shown to modify specific sites of the Aβ peptide thus enhancing Aβ aggregation and its neurotoxicity. Following Aβ modification at Lys-16, peptide aggregates were formed faster than in the case of modification at Lys-28 or at Asp-1 (Usui et al., 2009).

Further, evidence has emerged that Aβ has predominant cholesterol oxidase activity, particularly in the presence of divalent cations such as Cu^2+^. Significantly elevated levels of 4-cholesten-3-one were reported in brains of Aβ transgenic mice and in brain tissue of AD patients (Puglielli et al., 2005; Yoshimoto et al., 2005).

The interaction of Aβ with cell membranes is the crucial event in AD pathogenesis. Of note, there is less evidence to date on the negative effects of oxysterols on Aβ binding to the cell membranes. Because the orientation of oxysterols in the cell membrane differs from that of cholesterol, they are less able to condense lipids, thus modifying some physical properties of membrane, including raft domains; it has thus been suggested that oxysterols may facilitate Aβ interaction with cell membranes. The effects of 7-K and 7β-OH on enhancing Aβ insertion into the lipid bilayer, by decreasing intermolecular cohesive interaction, have been demonstrated (Kim and Frangos, 2008). Using a model membrane, it was shown that 25-OH and 7-K render the membrane more sensitive to Aβ, in contrast to the role played by cholesterol, which inhibits Aβ’s interaction with membranes. 7-K facilitated Aβ’s localization in the membrane, while 25-OH stimulated the peptide’s insertion but lead to membrane modification. In addition, the higher potential of Aβ_1–42_, compared to Aβ_1–40_, to interact with the membrane has also been demonstrated (Phan et al., 2013). Further, it is hypothesized that increased oxysterol concentrations, mainly of 7-K, but also of 24-OH and β-EPOX, may enhance exocytosis and neurotransmitter release in damaged areas of the brain, thereby aggravating neuronal excitotoxicity (Ma et al., 2010). Further, in our study we observed that 24-OH, 27-OH, and 7β-OH markedly enhanced the binding of Aβ_1–42_ on membranes of human differentiated neuronal cell lines (SK-N-BE and NT-2), by up-regulating CD36 and β1-integrin receptors (Gamba et al., 2011), two components of the multireceptor complex CD36/β1-integrin/CD47, through which Aβ peptide binds to cell membranes (Verdier et al., 2004; Yu and Ye, 2015). This event might favor the accumulation of the toxic Aβ_1–42_ peptide into neurons.

Although oxysterols have been analyzed for their involvement in neurotoxicity and Aβ production during AD progression, their role as natural ligands for LXR is now emerging (e.g., 24-OH, 27-OH, 22-OH, 25-OH, 4β-OH, and 7α-OH) (Vaya and Schipper, 2007). Indeed, astrocytes are sensitive to 24-OH-mediated up-regulation of ApoE, a LXR-target gene involved in cholesterol efflux (Abildayeva et al., 2006). Moreover, it has been reported that 27-OH prevents Aβ generation from primary human neurons, not by modulating α-, β-, or γ-secretase, but rather by overexpressing LXR-responsive genes (*ABCA1*, *ABCG1* and *ApoE*) (Kim et al., 2009b). Moreover, incubation of primary brain cells with 22-OH significantly reduced Aβ secretion in a dose-dependent manner, while ABCA1 expression and cholesterol efflux were induced (Koldamova et al., 2003).

Recent *in vitro* evidence also suggests that 24-OH and 27-OH might contribute to decreasing the influx of Aβ peptide into the brain across the BBB, increasing expression of the ABCB1 transporter in brain capillary endothelial cells, resulting in protection from peripheral Aβ entry (Saint-Pol et al., 2013). Of note, ABCB1 has never been described as an LXR target gene, and other nuclear receptors might control its transcription. Conversely, treatment of brain pericytes with 24-OH up-regulated ABCA1 expression that was correlated with an increase of cholesterol efflux, whereas 24-OH treatment did not reduce the pericytes’ ability to accumulate Aβ in the cells (Saint-Pol et al., 2012). The clearance of Aβ might also be mediated through microglia-induced phagocytosis of Aβ, which depends on LXR activation (Terwel et al., 2011).

Of note, LXR activation not only regulates cholesterol homeostasis, and Aβ peptide transport and clearance, but also neuroinflammation. Studies have shown that LXR activation inhibits inflammatory gene expression, pointing to the ability of LXRs to inactivate promoters of pro-inflammatory genes (Wang et al., 2002; Cao et al., 2007; Zelcer et al., 2007; Sodhi and Singh, 2013; Steffensen et al., 2013). Moreover, LXR activation may prevent neuroinflammation, by indirectly down-regulating TLR target genes. However, although LXR-activating oxysterols might reduce membrane cholesterol content and inflammation, they may also activate opposing pathways, and induce inflammation independently of LXRs. In our very recent study, we observed that 27-OH, 24-OH, and 7β-OH enhanced inflammatory molecule expression in human neuroblastoma SH-SY5Y cells via TLR4/cyclooxygenase-2/membrane bound prostaglandin E synthase; this clearly indicates that oxysterols may promote neuroinflammation in AD (Testa et al., 2014).

Although it can be assumed that oxysterols may increase the activation of microglia promoting their phagocytosis, there is less evidence to date on their effects on microglial phagocytosis during neuroninflammation. The phagocytosis of fibrils and large aggregates of Aβ by microglia is an important neuroprotective mechanism for Aβ peptide clearance (D’Andrea et al., 2004; Colton and Wilcock, 2010) but, in later stages of AD, the increased inflammatory molecule release makes the microglia phagocytically inactive leading to neuronal death (Hickman et al., 2008; Krabbe et al., 2013). Among the receptors promoting Aβ phagocytosis and clearance by microglia, the CD36 scavenger receptor appears to be involved and its increased expression may be crucial in preventing AD (Verdier et al., 2004; Yu and Ye, 2015): CD36 initiates a signaling cascade that promotes microglial activation and recruitment to β-amyloid deposits in the brain (Stuart et al., 2007). Concernig sterols, it has been shown that cholesterol (20 μM) and α-EPOX (20 μM) do not interfere with CD36 membrane distribution but both compounds were found to up-regulate the total CD36 levels in the mouse microglial cell line BV-2 potentiating phagocytosis in LPS-stimulated cells (Račková, 2013). Moreover, treatment with methyl-β-cyclodextrin, a reagent able to remove cholesterol from cell membranes, inhibited phagocytosis in LPS-activated microglia, indirectly supporting the potential role of sterols in phagocytosis (Churchward and Todd, 2014).

## Conclusion

This review has pointed up the vicious circle connecting oxidative stress and inflammation in AD. Alongside oxidative stress and neuroinflammation, altered cholesterol metabolism in the brain and hypercholesterolemia also significantly contribute to AD pathogenesis. Thanks to consistent research evidence, it is now believed that oxidized cholesterol is the driving force behind the development of AD and that oxysterols are the link connecting altered cholesterol metabolism and hypercholesterolemia to this neurodegenerative disease. Oxysterols play a fundamental role, by enhancing inflammation, Aβ generation and accumulation, and neuron death.

The involvement of oxysterols in AD pathogenesis, and the analysis of such products in the plasma and CSF, may contribute to clarifying the role of cholesterol metabolism in AD; ultimately, it may be helpful in developing therapeutic strategies to prevent or slow AD pathogenesis.

## Conflict of Interest Statement

The authors declare that the research was conducted in the absence of any commercial or financial relationships that could be construed as a potential conflict of interest.

## Abbreviations

α-EPOX: 5α, 6α-epoxicholesterol
β-EPOX: 5β, 6β-epoxicholesterol
24-OH: 24-hydroxycholesterol
25-OH: 25-hydroxycholesterol
4β-OH: 4β-hydroxycholestrerol
7β-OH: 7β-hydroxycholesterol
7α-OH: 7α-hydroxycholesterol
7-OH-4-C: 7α-hydroxy-3-oxo-4-cholestenoic acid
7-K: 7-ketocholesterol
ABCA1: ATP-binding cassette transporter A1
ABCG1: ATP-binding cassette transporter G1
AD: Alzheimer’s disease
ApoE: apolipoprotein E
APP: amyloid precursor protein
Aβ: amyloid β
BACE1: beta-site amyloid precursor protein cleaving enzyme 1
BBB: blood-brain barrier
CNS: central nervous system
CSF: cerebrospinal fluid
CYP27A1: 27-hydroxylase
CYP46A1: 24-hydroxylase
LPS: lipopolisaccaride
LXR: liver X receptor
NF-κB: nuclear factor-κB
NFT: neurofibrillary tangles
NO: nitric oxide
PSEN1: presenilin 1
PSEN2: presenilin 2
RNS: reactive nitrose species
ROS: reactive oxygen species
TLR: Toll-like receptor.
